# A Potential Role of *Salmonella* Infection in the Onset of Inflammatory Bowel Diseases

**DOI:** 10.3389/fimmu.2017.00191

**Published:** 2017-02-28

**Authors:** Bárbara M. Schultz, Carolina A. Paduro, Geraldyne A. Salazar, Francisco J. Salazar-Echegarai, Valentina P. Sebastián, Claudia A. Riedel, Alexis M. Kalergis, Manuel Alvarez-Lobos, Susan M. Bueno

**Affiliations:** ^1^Facultad de Ciencias Biológicas, Departamento de Genética Molecular y Microbiología, Millennium Institute on Immunology and Immunotherapy, Pontificia Universidad Católica de Chile, Santiago, Chile; ^2^Facultad de Ciencias Biológicas y Facultad de Medicina, Departamento de Ciencias Biológicas, Millennium Institute on Immunology and Immunotherapy, Universidad Andrés Bello, Santiago, Chile; ^3^Facultad de Medicina, Departamento de Endocrinología, Pontificia Universidad Católica de Chile, Santiago, Chile; ^4^INSERM, UMR 1064, Nantes, France; ^5^Facultad de Medicina, Departamento de Gastroenterología, Pontificia Universidad Católica de Chile, Santiago, Chile

**Keywords:** inflammatory bowel disease, Crohn’s disease, ulcerative colitis, gut microbiota, *Salmonella enterica* serovar Typhimurium, innate immune response, virulence factors

## Abstract

Inflammatory bowel disease (IBD) includes a set of pathologies that result from a deregulated immune response that may affect any portion of the gastrointestinal tract. The most prevalent and defined forms of IBD are Crohn’s disease and ulcerative colitis. Although the etiology of IBD is not well defined, it has been suggested that environmental and genetic factors contribute to disease development and that the interaction between these two factors can trigger the pathology. Diet, medication use, vitamin D status, smoking, and bacterial infections have been proposed to influence or contribute to the onset or development of the disease in susceptible individuals. The infection with pathogenic bacteria is a key factor that can influence the development and severity of this disease. Here, we present a comprehensive review of studies performed in human and mice susceptible to IBD, which supports the notion that infection with bacterial pathogens, such as *Salmonella*, could promote the onset of IBD due to permanent changes in the intestinal microbiota, disruption of the epithelial barrier and alterations of the intestinal immune response after infection.

## Introduction

Inflammatory bowel disease (IBD) is defined as a set of pathologies that exhibit a progressive and chronic phenotype, where the intestinal immune response and the normal gut microbiota are altered (1). IBD usually begins in adolescence and persists lifelong (2). The symptoms of these inflammatory disease are not only limited to the gastrointestinal level but also produces systemic complications such as fever, weight loss, delayed sexual maturation and growth, among others. Further, extraintestinal diseases can be associated with IBD, including arthritis (3). The most common clinical manifestations of IBD are Crohn’s disease (CD) and ulcerative colitis (UC) (2). CD, a manifestation that affects females in a greater proportion, is characterized by a chronic and transmural inflammation, specifically at the colon and small intestine. However, inflammatory lesion during CD can be found at any section of the gastrointestinal tract, from the mouth to the anus (4). These lesions can affect all layers of the gastrointestinal tract, producing strictures and fistulae (5, 6). CD mostly affects the young population, with a peak of incidence in the early adulthood (between 20 and 30 years old). UC, on the other hand, is a manifestation more common in males and only affects the superficial layer of the colon, with a continuous inflammation comprising from the rectus to variable distances along the intestine (6, 7). The incidence of IBD is higher in industrialized or westernized regions, such as the United States and Northern and Western Europe, and it is mild in South America and Africa (8). The same pattern is observed in urban and rural areas, indicating that industrialization can be considered as an etiological factor for major incidence of IBD (7, 8). Additional factors, such as environmental and genetic factors, interact to determine the onset and development of disease.

Different studies have shown the correlation between environmental and genetic factors that could lead to a dysfunction of the intestinal epithelial barrier, with a consequent deregulation in the function of the mucosal immune cells. These alterations lead either to an inappropriate recognition of the gut microbiota or an increased susceptibility to infections (7, 9). Moreover, environmental factors can differentially affect predisposition of individuals, by increasing their susceptibility to develop IBD (10). The increase in the incidence of IBD has been associated with several factors common to modern lifestyle, such as use of antibiotics, vaccines, contraceptives, vitamin D status, and better hygiene. Further, changes associated with westernization, such as high consumption of fats, refined sugar, and carbohydrates, have also been implicated in the incidence increase for these diseases during the last decades (11). According to previously observed associations between the consumption of some food and incidence of UC or CD, it is presumed that the diet could induce changes in the microbiota composition and in the cellular adhesion to the intestinal barrier (12), which could in turn lead the development of IBD (11).

Inflammatory bowel disease is a disease highly influenced by genetic factors. Several genetic mutations and polymorphism have been described in both UC and CD (13–15). Interestingly, some of the polymorphisms associated with IBD locate in genes encoding proteins involved in bacteria recognition, degradation, or translocation through the intestinal epithelial barrier. For instance, it has been described that in both UC and CD patients there are polymorphisms in genes associated with the *Th1/Th17 pathway* [*il23r* (16), *il12b* (17), or *stat3* (17) genes]; *autophagy* [*atg16l1* (18), *irmg* (19), and *nod2* (20)]; and *epithelial barrier* [*jak2* (13) and *il-10* (14)]. These mutations affect the capacity of the innate immune cells to handle intracellular bacteria due to an aberrant autophagy process. These alterations result in a response unable to control systemic bacteria spread, which predisposes the host to an increased pathogen colonization and an enhanced susceptibility to these diseases (21). Another gene involved in CD is *nod2*, which encodes the nucleotide-binding oligomerization domain-containing protein 2 (NOD2). This gene is in chromosome 16 and encodes an intracellular receptor for the muramyl dipeptide (MDP), a component of bacterial cell wall. When this gene is silenced, it is observed that an aberrant IL-1β production occurs in response to bacterial endotoxins, which leads to an impaired early immune response (22, 23). Mutation in *nod2* also affects the function of Paneth cells, diminishing the production of α-defensin, an antimicrobial peptide secreted by this type of cells (24, 25). Variants of this gene, combined with polymorphisms in *tlr9* or *atg16l1*, increase the risk of suffering IBD (22). The *atg16l1* gene encodes a key protein in the process of autophagy (9), which is required for a proper innate immune response against microorganisms. Mice ATG16L1^HM^, which carry a disruption of the *atg16l1* gene with a concomitant decreased protein level, display abnormal Paneth cells function due to a defect of the granule exocytosis pathway. Such a phenotype can also be observed in CD patients. In consequence, the secretion of lysozyme is altered, and the expression of genes involved in injury response is increased (26). These data indicate that Paneth cells have a unique sensitivity to autophagy gene disruption, which lead to endoplasmic reticulum stress in the intestinal epithelium. Thus, the autophagy process could have a specific role in these cells, as it seen in CD patients (26–28). In another study, researchers found that patients with variants in *nod2* and/or *atg16l1* genes display an increased secretion of TNF-α in response to bacterial translocation through the intestinal epithelial barrier, which is directly related to the aggravation of intestinal inflammation, disease activity, and relapsing episodes (22). Through genetic analyses, it was shown that CD has more genetic components (such as loci associated with susceptibility to the disease) than does UC (21). Therefore, a better knowledge of the genetic variables and their interaction with environmental factors will generate a breakthrough in pharmacogenomics, which could be used as a treatment for the disease, improving the tolerability and effectiveness of the therapies used nowadays (21).

## Contribution of the Intestinal Epithelial Barrier to Bacterial Infections and IBD Development

**Figure 1:**
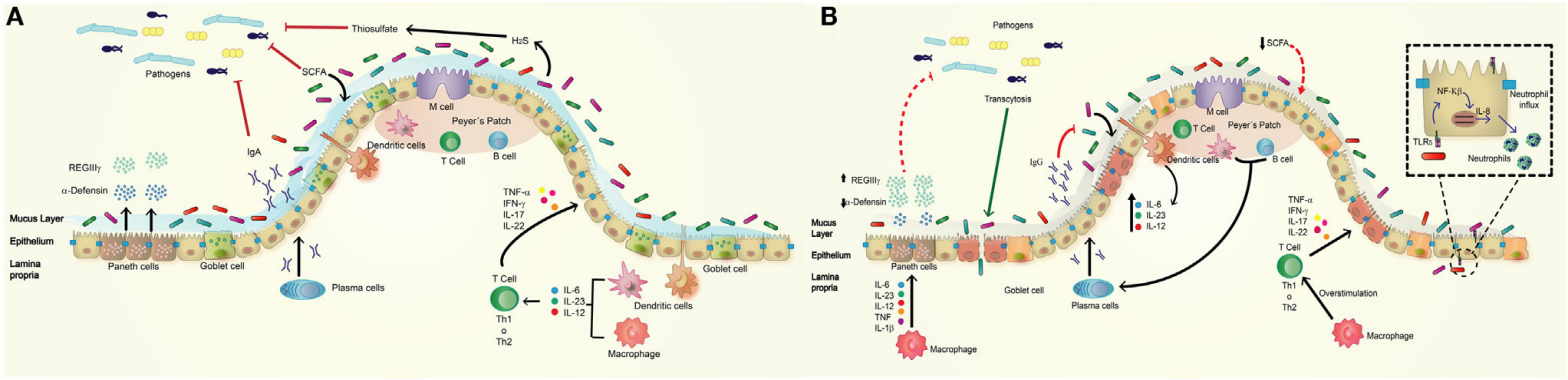
**Normal intestinal epithelium versus altered intestinal epithelium observed in inflammatory bowel disease (IBD)**. **(A)** The normal intestine presents a high secretion of bactericidal molecules (defensins, REGIIIγ, and IgA) as mechanisms of defense against pathogenic bacteria. The commensal microbiota inhibits the access of pathogens to the epithelial barrier by competing for nutrients, maintaining homeostasis of the epithelial barrier, and supporting the host immune response. The commensal microbiota is composed of mainly *Firmicutes* and *Bacteroidetes*, and a lower percentage of *Proteobacteria* and *Actinobacteria*. They promote the secretion of mucus and antimicrobial peptides [short-chain fatty acid (SCFA), H2S] and the activation of some pathway of immune system such as the activation of macrophages and dendritic cells in lamina propria, and the production of cytokines such as IL-6, IL-23, and IL-12, which activates Th1 or Th17 cells to produce cytokines acting on intestinal epithelium. **(B)** The intestine of IBD patient has a deregulated response to commensal microbiota by a decrease in the secretion of antimicrobial peptides such as α-defensins and increase in REGIIIγ as compensatory effect; these effects have relation with defect in Paneth and goblet cells. IBD patients showed lower *Firmicutes* and *Bacteroidetes* but have increased amounts of *Proteobacterias* resulting in a decrease production of SCFA, mucus, and increased inflammation. The epithelium produces an abnormal amount of IgG against commensal microbiota instead IgA. Macrophages produce higher amounts of cytokines that overstimulate Th1 or Th17 cells, which secrete pro-inflammatory cytokines to epithelium.

The intestinal epithelial barrier physically separates the intestinal lumen from deeper layers, such as the lamina propria (29). It is organized in crypts and villi and composed of four types of specialized cells: *absorptive enterocytes* that have metabolic and digestive functions and can also secrete some antimicrobial peptides; *goblet cells*, specialized in mucus secretion; *enteroendocrine cells* that secrete hormones; and *Paneth cells* that mostly secrete antimicrobial peptides into the crypts of the small intestine (Figure 1A) (29). *M cells*, which are specialized follicle-associated epithelial cells that cover Peyer’s patches, are found in the small intestine. The function of these cells is sensing luminal content, a task required for the correct functioning of the epithelial barrier. Antigens and microbes captured by M cells are transported across the epithelial barrier and presented to immune cells residing in the lamina propria, through a process denominated transcytosis, which is essential for antigen-specific mucosal immune response (Figure 1A) (30).

Through the intestinal barrier, there are several proteins that monitor the environment of the intestinal lumen. Pattern-recognition receptors (PRRs), for instance, are proteins expressed by different type of innate immune cells located in the intestinal tissue, such as dendritic cells, phagocytic macrophage and granulocytes, cytotoxic natural killer, and γδ-T cells; as well as intestinal epithelial cells. PRRs sense pathogens through pathogen-associated molecular patterns (PAMPs), such as LPS or flagellin (31, 32). PRRs also have a role in the regulation of intestinal epithelial barrier, repair, and immune homeostasis (33, 34). The PRRs include different type of members: toll-like receptors (TLRs), NOD-like receptors (NLRs), RIG-I-like receptors (RLRs), and C-type lectin receptors (35, 36). RLRs and C-type lectin receptors are mainly involved in viral and fungal recognition, respectively (35). TLRs are present in the surface of epithelial cells and endosomes, and even some can be found inside Paneth cells or enteroendocrine cell (35). TLRs are distributed in different portions of the intestine and differentially expressed on the apical or basolateral side of the cell. Then, the same PRR may respond differentially depending on its localization. For instance, TLR5 only produces an inflammatory response if it binds flagellin in the basolateral surface of the epithelial barrier, which involves the transcription of pro-inflammatory cytokines (37). Other proteins involved in pathogen recognition at the intestine are NLRs, which are found in the cytosol of macrophages, dendritic cells, and Paneth cells. These intracellular proteins sense PAMPs as well as endogenous molecules released from damaged cells, called damage-associated molecular patterns. NLRs can sense a distinctive substructure from peptidoglycan of mostly all Gram-negative and Gram-positive bacteria (35, 38). Among the NLRs, NOD1 and NOD2 are the most studied. Both recognize products released by division of intracellular bacteria and, after NOD2 is activated, NF-κB translocates to the nucleus and allows the production of IL-6, IL-1β, and TNF-α, to trigger a pro-inflammatory response (39).

M cells have another type of receptor, the glycoprotein-2 receptor (GP2), which is found in the apical side of epithelial barrier and serves as transcytosis receptor for many antigens derived from commensal and pathogenic bacteria. This endocytic receptor recognizes FimH, a component of type I pilus of diverse enterobacteria, such as *Escherichia coli* and *Salmonella* Typhimurium. GP2 interacts with bacterial pilus proteins, allowing the capture of the bacterium by the cell and its transport across the barrier to the Peyer’s patches and other gut-associated lymphoid tissues (29, 30).

Another important component of the intestinal barrier is a wide assortment of resident microbial communities that prevent infection by competing with pathogenic bacteria. For example, commensal bacteria produce secondary metabolites that specifically inhibit members of the same or similar species able to cause infection. It has been shown that there are two bacterial phyla that predominantly reside in the gut of healthy individuals; these are *Bacteroidetes* (Gram-negative) and *Firmicutes* (Gram-positive) (40). It has been reported that microbial communities may change due to age, nutrition, inflammatory processes, and gastrointestinal disease (Figure 1A) (41), and it has been demonstrated that the presence of commensal microbiota induces a basal expression of certain TLRs (such as TLR2 and TLR5), as compared to the basal levels of expression observed in specific-pathogen free mice and germ free mice (42). The same report shows that microbiota is essential to trigger a proper inflammatory response to infection by some pathogenic bacteria (42). Thus, a complex interplay between the host immune system and the microbiota is required for gut microbiota homeostasis. For instance, the production of IL-6 and TNF-α is triggered by commensal microbiota, and a proper functioning of TLR is required for protection against injuries of the intestinal epithelium. Thus, it is possible that the use of antibiotics in pharmacological dose could impair the production of these cytokines due to a reduction of commensal microbiota, which in turn might result in a reduced tissue repair ability (33).

A defect in the functioning of any of the epithelial barrier components mentioned above leads to an aberrant inflammatory response and promotes susceptibility to some diseases, such as IBD. In this case, it is known that defects in the response of goblet and/or Paneth cells generate a type of colitis or a spontaneous inflammation that resembles CD (29). Further, the dual role of TLRs could be also an important factor in the development of the disease (33, 42, 43).

## Defects in the Immune Response to Bacterial Pathogens and Microbiota Described in IBD Patients

As discussed above, the gut is constantly exposed to commensal microbiota and foreign microorganisms introduced by food consumption. The immune system helps to keep the correct homeostasis between the immunosuppressive response—which prevents overreaction to harmless antigens—and the protective response against pathogens (44). Intestinal tissue is highly deteriorated in IBD patients, especially in CD patients, due to an uncontrolled reaction of the immune system against bacterial antigens (44, 45). Thus, the epithelium is highly affected, and a pro-inflammatory response results in a loss of tolerance to the normal microbiota (Figure 1B) (45).

Several alterations in mechanisms of epithelial barrier protection are related to the pathology of patients with IBD, because a barrier dysfunction leads to impaired immune responsiveness, as in this disease. One of them is, for example, the secretion of mucus by goblet cells. IBD patients have decreased production and secretion of mucin-2, the main component of the mucus, which is related to the development of inflammation (46). This leads to a decreased protection of the epithelial barrier and a greater number of bacteria that are in direct contact with the epithelium (46, 47). Accordingly, mice lacking the gene encoding Muc2 (Muc2^−/−^) have increased gut inflammation and weight loss, and this deficiency could contribute to the onset and perpetuation of the colitis. Beside this, it is important to mention that the microbiota has an important role in regulating the secretion of colonic mucus (46, 48).

The process of epithelial regeneration after an injury is also affected in IBD patients. Epithelial repair processes are divided in two phases: the first one involves the re-distribution of the existing cells, a process that is regulated by TGF-β. In the second phase, cell proliferation is regulated by cytokines such as IL-6, secreted by pro-inflammatory lymphocytes (27). TGF-β levels are increased in active UC and CD patients, relative to control patients, due to a constant inflammatory process and injury of the epithelial barrier that must be repaired (49). IL-6 is induced early after injury, allowing proliferation of intestinal epithelial cells that is needed for a proper healing of the epithelium but also has functionality in tumorigenesis and chronic inflammation. These functions may be related to the development of cancer in IBD patient, having a central role in the pathophysiology of the disease (50–52). Although the blockage of these pathways did not improve the histopathological score, it does improve disease activity score, and it may have a therapeutic potential (53).

As previously discussed, the epithelial barrier must be functional and not allow the entry of pathogens to the inner layers. If the tight junctions are altered, the permeability of the barrier could increase and, along with this, there will be a greater paracellular flow of microorganisms, promoting the infection of the lamina propria with pathogenic and/or opportunistic bacteria (54). It has been described that both TNF-α and IFN-γ can modify these junction structures, and it is known that IBD patients have an elevated production of TNF-α, which could be mediating the increased permeability due to the loss of tight junctions structure (54). However, it is not well understood if this dysfunction is a consequence of increased inflammation during an active disease or if it is the cause of IBD development, because some susceptible patients without symptoms or those in remission also show altered intestinal permeability (55). In normal conditions, the intestine is the major antibody producer tissue of the body, and the intestinal mucous membrane contains more than 80% of the activated B cells (56). IBD patients have a dysfunction in the B cell response, which involves an abnormal mucosal secretion of IgG antibodies against commensal bacteria instead of the physiological secretion of IgA (Figure 1B) (57, 58). This overproduction causes an exacerbated pro-inflammatory response and injury in the epithelium, which is not observed in healthy individuals and may be relevant in the development of the disease (59). Further, IBD patients also present antibodies to self-antigens or cross-reactivity against several bacterial and fungal antigens, which often precedes the onset of the disease (60). For example, CD patients have antibodies against *Saccharomyces cerevisiae* (ASCA), *E. coli, Pseudomonas fluorescens*, and flagellin (61), which are directly related to the aggravation of the disease. Overreaction to these bacterial antigens generates additional clinical manifestations, such as stenosis and internal perforations.

## Effect of Antibiotic Treatment in IBD Patients

Inflammatory bowel disease is characterized by an augmented bacterial density at the mucosal level (62, 63), as well a diminished number of anti-inflammatory commensal bacteria, such as the Gram-positives *Firmicutes* and *Actinobacteria* (56, 64). As a consequence of this dysbiosis (dysregulation of commensal microbiota), an increased number of potentially harmful bacteria (such as Enterobacteriaceae) can occur, producing inflammation (65). For these reasons, maintaining a proper ratio of these populations is highly relevant, because they constitute a barrier against pathogenic bacteria (56). In support of this idea, studies have shown that UC patients, even in remission, have dysbiosis when compared to controls, with increased numbers of opportunistic pathogens such as *Campylobacter* spp. and *Helicobacter* spp. (66, 67). Further, these patients show a reduction in the number of the cluster related to the metabolism of short-chain fatty acids (SCFAs), which generates less anti-inflammatory environment in their guts (66). For these reasons, the use of antibiotic in IBD patients to treat septic complication, such as abscesses and wound infection, is still conflicting because it decreases the number of intestinal bacteria and alters the normal composition of the microbiota (68). Moreover, treatment with antibiotics increases the susceptibility of the patient to acquire an infection by *Clostridium difficile* (68, 69).

Along the same line, some studies have shown that antibiotic therapy is functional in UC and in CD, and the therapy works better if given orally. Two meta-analysis supports the role of the antibiotics in induction of the remission of IBD (70), specifically UC (71). This observation agrees with a study performed in some pediatric patients with severe refractory UC, where the children receive a triple therapy for 2–3 weeks with amoxicillin, metronidazole, and doxycycline in children over 7 years of age. This study shows that the treatment induced remission in 47% of the patients and the effects observed depend on the physiological characteristics of the patients and the current treatment used to ameliorate the symptoms (69). A systematic review shows that the induction of remission can occur in both CD and UC patients, but that is still not sufficient information to recommend a type or a cocktail of antibiotic to treat effectively the disease (72). In summary, most of the processes described above, which can promote the onset and severity of IBD, are related to proper bacterial location and clearance in the intestine. In the next section, we will discuss how pathogenic bacterial infection could trigger IBD in susceptible individuals.

## *Salmonella enterica* Infection and IBD

Many pathogenic microorganisms have been implicated in the exacerbation or development of IBD (73): *Campylobacter* (1), *E. coli* (74), *Helicobacter pylori* (75), *Mycobacterium avium* subspecies *paratuberculosis* (76), and *C. difficile* (77). We will focus on *S. enterica* serovar Typhimurium (1).

*Salmonella enterica* serovar Typhimurium (*S*. Typhimurium) is a facultative, Gram-negative and intracellular bacterium, which infects several host including humans (78). *S*. Typhimurium can cause a severe inflammation of the intestinal mucosal epithelium, resulting in humans, gastroenteritis, and in mice, typhoid-like systemic illness (78). As every pathogen, *S*. Typhimurium has several virulence genes, located in at least five *Salmonella* pathogenicity islands (SPIs), which are genetic elements within the *Salmonella* chromosome that was acquired probably by horizontal gene transfer (79). The most important and more studied SPIs are SPI-1 and SPI-2. Both SPIs encode type III secretion systems (T3SS). These are complex machineries formed by more than 20 proteins that allow contact-dependent translocation of a set of different effector proteins into the eukaryotic cytoplasm (80, 81). SPI-1 allows *Salmonella* to invade epithelial cells, while SPI-2 allows the survival and replication inside phagocytic cells (79, 82).

The first step in *S*. Typhimurium infection is to cross the intestinal epithelial barrier, which can be accomplished through four different routes (83, 84). The main route is through the activation of virulence factors encoded in SPI-1. A second route of invasion requires the rupture of tight junctions of the epithelial cells, which changes the basal permeability of the intestinal barrier (85). Finally, there is another entry through CX3CR1^+^ DCs (86) interleaved in the epithelial barrier, reaching the bloodstream to spread to extraintestinal sites, through the transport in CD18^+^ phagocytes (87).

**Figure 2:**
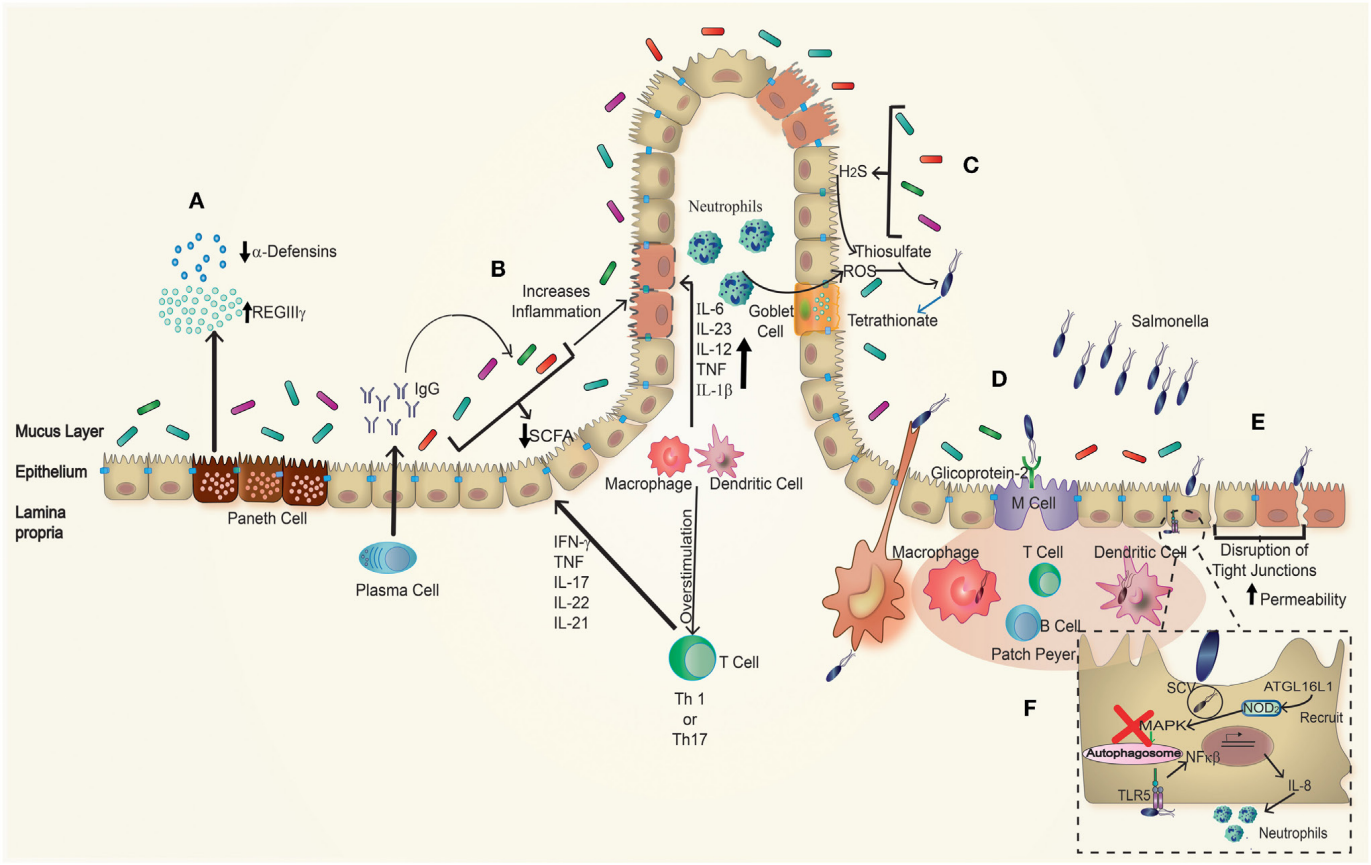
**Effect of *Salmonella* infection in inflammatory bowel disease (IBD) patients**. In genetically susceptible to IBD patients, many parameters are disrupted. **(A)** Paneth cells have an impaired secretion of antimicrobial peptides showing a decrease in the amounts of α-defensins, as well as increased amounts of REGIIIγ, which is associated with impaired protection against pathogens. **(B)** Plasma cells have a polarized antibodies’ secretion to the production of IgG antibodies targeting the individual’s own microbiota. Beside this, the proportions of commensal microorganisms present are unbalanced related to a healthy host. Due to this imbalance, there is a decrease production of short-chain fatty acid (SCFA), which generates an increase in inflammation. Taken together, these effects generate an environment more vulnerable to further infection. Furthermore, **(C)** *S*. Typhimurium recruits neutrophils to the lumen, which generates ROS, producing tetrathionate in the intestinal lumen; this compound is used by *Salmonella* as electron acceptor, which gives advantage to *S*. Typhimurium over the microbiota. **(D)** *Salmonella* infection is produced by the entry through DCs interspersed in the epithelial barrier or M cells that recognize it through glycoprotein-2, accessing to the Peyer’s patches. **(E)** *Salmonella* can also get into the epithelial cells forming membrane ruffling or through disruptions of tight junctions caused by itself and in this case especially in inflamed epithelium of IBD patients. **(F)** In the basolateral side, *Salmonella* can be recognized by TLR5 stimulating an increased production of NF-κβ, which correlates with an enhanced recruitment of neutrophils, finally once within the epithelial cells, *Salmonella* blocks the autophagosome pathway avoiding its own degradation.

The second step in the cycle of infection of *S*. Typhimurium is the expression of T3SS-2 inside immune cells, such as macrophages and DCs of the Peyer’s patches and lamina propria (88), in which this bacterium can survive and replicate within a specific compartment known as *Salmonella*-containing vacuole (SCV), which avoids lysosomal degradation and antigen presentation (83, 84). This initial invasion of the Peyer’s patches leads to an inflammatory response with recruitment of immune cells, mainly neutrophils, which should prevent bacterial dissemination (Figure 2D).

## Immune Response Against *Salmonella*

Commensal microbiota, mucus layers, antimicrobial peptides, and tight junctions work together to maintain the integrity of the epithelial cellular barrier and prevent infection of pathogenic bacteria (89). Despite all these defense mechanisms, *S*. Typhimurium can modify tight junctions to increase the permeability of the barrier, which allows its translocation through the epithelial cell monolayer, due to the secretion of a protein-denominated AvrA through the T3SS-1 (85). This protein impaired the activation of pro-inflammatory cytokine, such as IL-6 (90) and can affect cellular proliferation activating the β-catenin pathway (91). Beside this, AvrA can modulate the c-Jun N-terminal kinase, which suppresses the apoptosis process during early steps of the infectious process (92). All these mechanisms together allow *S*. Typhimurium to produce inflammation of the intestine, without destroying the epithelium.

Toll-like receptors and NLRs recognize various compounds of *S*. Typhimurium, activating pathways associated with a pro-inflammatory response such as pyroptosis, which is a cell death response that involves the production of caspase-1, required for the secretion of mature IL-1β and IL-18 by macrophages (93, 94). For example, flagellin is recognized by TLR5 in the basolateral surface (30, 32, 37), which actively promotes the production of pro-inflammatory cytokines, such as IL-6 (95) and IL-8 (96), through the activation of the transcription factor NF-κB and MAP kinases, followed by the recruitment of inflammatory cells and the activation of the adaptive immune response. Although immune cells rapidly clear bacteria, an important fraction disseminates to deeper organs using phagocytic cells as a “Trojan horse.” Using this mechanism, *S*. Typhimurium can migrate from the site of infection to the lymph nodes and activate T cells (97). In addition, *S*. Typhimurium can induce the production of caspase-1 *via* Nlrc4 (a type of NLR) through the recognition of flagellin, which enters through the T3SS-1 to the cytosol of phagocytic cells. This cytosolic response is independent of the one generated by TLR5 (extracellular) (36). In this way, *S*. Typhimurium uses this defense mechanism (production of inflammasome and pyroptosis process) to spread to other immune cells and disseminate systemically from the gastrointestinal tract (93).

During an infection with *S*. Typhimurium, the response of NOD1 or NOD2 occurs through the activation of the transcription factor NF-κB, which has an important role in the regulation of cytokine production. Thereby, it modulates the transmigration of neutrophils to the source of infection and thus the intensity of the inflammation (Figure 2F). The activation of another signaling cascade *via* NOD2, triggered also by MDP, has a direct relation with the activation of the inflammasome NLRP3 through NF-κB, caspase-1, and the consequent secretion of IL-1β and IL-18 (98, 99). The correct function of these receptors is important, because through its activation they interact directly with the ATG16L1 protein and lead to the proper initiation of the autophagy process (99, 100).

All these effects produce an increase in inflammation after bacterial infection because of the structure loss of the intestinal mucosa, producing diarrhea with concomitant loss of liquid and electrolytes (97). This response against foreign microorganisms must be controlled to reduce tissue damage or to prevent a systemic infection, as it could be the case of *S*. Typhimurium (Figure 2B). However, sometimes the immune response is altered and generates an overreaction against its own components, as in the case of IBD.

## *Salmonella* Interaction with Microbiota

Commensal microbiota is mostly fermentative and produces at least three SCFAs, which are mainly acetate, propionate, and butyrate (101). The concentration and distribution of these compounds vary along the gut and exert different effects on colonization of pathogenic bacteria, such as *Salmonella*. In the ileum, there are higher concentrations of acetate, which induce the expression of genes within SPI-1, allowing the invasion of the ileum (101). Moreover, propionate and butyrate are present in higher amounts in colon and cecum (102) playing an antimicrobial effect, diminishing the expression of the same invasion genes (101–103). Therefore, SCFAs produced by the microbiota influence *S*. Typhimurium’s “choice” of the site of colonization, and given that any change in the composition of the microbiota will vary the proportion of these compounds, they could allow for a different disease phenotype.

*S*. Typhimurium infection produces the transmigration of neutrophils, which oxidize the endogen sulfur compound thiosulfate $\left({\text{S}}_{2}{\text{O}}_{3}^{2-}\right)$ in the intestinal lumen, generating tetrathionate $\left({\text{S}}_{4}{\text{O}}_{6}^{2-}\right)$ (Figure 2C) (104, 105). This product is an electron acceptor for *S*. Typhimurium energetic processes and allows the utilization of ethanolamine as a nutrient by the bacterium. This is a competitive advantage over the fermentative bacteria from the microbiota, which are unable to use this product, so *S*. Typhimurium will overgrow and disseminate (41, 105, 106). Beside this, neutrophils induce the change of microbiota during an infection with *S*. Typhimurium secreting a serine protease (elastase), which has a direct effect to the microbiota (107). This way, *S*. Typhimurium used both the inflammation and the secretion of elastase to create a more favorable environment for its own colonization. *S*. Typhimurium can take advantage of the inflammatory response and promote its own growth and dissemination into host tissues. Moreover, this pathogen could play an important role in changing the microbiota composition in genetic susceptible individuals or patients with a chronic inflammation, such as IBD, due to mainly the dysbiosis in these patients (41, 108).

## *S*. Typhimurium Infection: Previous or after IBD Onset?

In this section, we will discuss some factors that could relate the intestinal inflammation that occurs in IBD and the intestinal infection caused by *S*. Typhimurium (Figure 2). A previous study suggests a connection between *S*. Typhimurium infection and IBD development (1). Furthermore, another study describes the presence of *Salmonella* and other enteropathogen toxins in the serum of IBD patients, which correlates to disease progression (109). However, a following study in Danish population suggested that increased detection of *Salmonella* and *Campylobacter* in stools of IBD patients is due to detection bias during the first year of infection (110). Although no association of exposure to *S. enterica* in CD patients was observed in a study made in another cohort of patient, a relation between CD, cigarette smoking, and anti-*Salmonella* antibodies in serum was observed (111). However, some studies suggest that during the course of IBD, due to the dysbiosis of the disease itself, the chances of an infection by enteropathogens are higher (108, 112). Other studies showing positive results with antibiotics treatment of IBD suggest the possibility of a pathogenic agent as the causative agent of the disease (72).

The permeability of the epithelium in IBD patients is altered and allows increased transcytosis of commensal and/or pathogenic bacteria, which could generate an inflammatory response. When an infection with an invasive bacterial pathogen (such as *S*. Typhimurium) occurs, it generates the recognition by basolateral TLR5 (Figures 2E,F), which initiates an inflammatory immune response (37). It has been shown that the signaling through TLR5 after flagellin recognition in a murine model of chemical-induced colitis generates an increase in the secretion of pro-inflammatory cytokines (32, 96) such as IL-8, which recruits neutrophils to the site of infection (113) and aggravates the clinical symptoms, producing severe histopathological damage in the colonic mucosa (32). Therefore, IBD patients can be more susceptible to an infection with a pathogen as *S*. Typhimurium, which could trigger the onset or aggravate the course of the disease, leading to a relapse. Additionally, *S*. Typhimurium synthesizes FimH, a protein that binds to M cells by interacting with GP2. GP2 has an epitope recognized by “anti-pancreatic” antibodies found in CD patients (114), which could be a consequence of infection with this pathogen or could involve the need for the combination between the receptor and FimH to produce antibodies.

A deregulation of the secretory function of Paneth cells has been observed in IBD patients. For instance, CD patients have diminished production of α-defensins at both mRNA and protein levels. Further, IBD patients have also an augmented secretion of Reg-lectin family members, such as RegIIIγ, which has a compensatory effect in the decrease in α-defensins (Figure 2A). This phenomenon is related to the increased adherence of the commensal microbiota to the enteric mucosa and an augmented penetration of the commensal bacteria to the mesenteric lymph nodes, which generates an inflammatory environment (115). All these impaired processes are being observed in the ileum of affected IBD patients and are more pronounced in individuals that also carry mutations in the *nod2* gene. However, these defects *per se* are not translated in increased inflammation of the intestine, which suggests that an additional trigger (such as bacterial infection) may promote IBD in these susceptible patients (116, 117). A recent study has described that the polymorphism T300A in the gene *atg16l1* results in a defective production of C-type lectin domain family 12 member A (CLEC12A) in CD patients, a protein potentially involved in antibacterial autophagy (118). This study shows that absence of CLEC12A prevents *S*. Typhimurium clearance by HeLa cells and that mice lacking CLEC12A are more susceptible to suffer a more severe infection (118). As mentioned above, mutations in the *atg16l1* gene also alter the function of Paneth cells due to changes in the granule exocytosis pathway (26). In the same context, *S*. Typhimurium diminishes the granules production and the secretion of lysozymes by Paneth cells, through the activation of p38/MAPK in the small intestine (117). This could be a survival mechanism of the bacterium and may be required by the subsequent infection process. Beside this, the infection activates a differentiation program that results in hyperplasia of Paneth cells in crypts, which could give as a result an acute inflammatory response and have some effect in the intestinal stem cells, due to an accelerated process of proliferation (119). Further, *S*. Typhimurium generates hyperplasia of Paneth cells through the activation of the Wnt pathway, but IBD patients have deficiencies in different factors (such as Tcf4, for example) of the same pathway, which is related to decrease the secretion of α-defensin (120) (Figure 2A). The combination of virulence factors displayed by *S*. Typhimurium and the genetic alterations of the host that prevent correct bacterial clearance suggests that an infection with *S*. Typhimurium in a susceptible host could generate changes in the proliferation and differentiation of Paneth cells, which in patients suffering IBD will exacerbate the defect produced by the mutation in some of the genes related to this pathology, which finally will modify the lysozyme secretion, with a consequent alteration in microbiota. All these changes generate a new or different niche to the infection and finally the onset of the disease with possible different phenotypes.

Other important factor related to IBD and *S*. Typhimurium infection is the formation of autophagosomes. *S*. Typhimurium survives inside the SCV without being recognized by the host cell. However, if this compartment is damaged, ubiquitination can modify the bacterium, leading to its autophagy encapsulation. Despite this, Ssel, a soluble protein secreted by T3SS, actively deubiquitinates the bacterium to prevent formation of the autophagosome (121). In this context, the intestinal epithelium responds to a *S*. Typhimurium infection with increased secretion of factors related to the autophagosome formation (121). Therefore, the activation of this pathway is one of the main processes required for resolution of the infection caused by this intracellular bacterium. In IBD patients, there are three genes related to the autophagosome process that are affected: *irgm, nod2*, and *atg16l1*, each associated with different grades of susceptibility to suffer IBD, which implies a defect in antigen uptake and its processing, the interaction between dendritic cells and intestinal epithelial cell (45), and the regulation of PRRs and inflammasome activation. These genes encode proteins that are important to contain the infection caused by *S*. Typhimurium (122).

Mutations in the *atg16l1* gene generate defects in the formation of autophagosomes, which implies that lower numbers of bacteria will be captured and so less efficient bacterial clearance will occur (121, 123). Beside this, patients with CD that carries an *atg16l1* mutation (45) have an impaired degradation of *S*. Typhimurium, making the host more susceptible to the infection. NOD2 is required for the formation and activation of the phagosome and for the recruitment of ATG16L1 to the site of entry of the bacterium, therefore mutations in this gene generate lower levels of autophagy and an impaired bacterial clearance (100). Mutation in the *atg16l1* gene impaired the correct antibacterial function of NOD2 in epithelial cells of the colon, and mutation in *nod2* generates an impaired signaling and bacterial killing, but this mutation only partially affects the autophagy process (124). So, in this case, an infection by *S*. Typhimurium in a susceptible host could be more severe, as proper function of the above mentioned proteins is required for the correct bacterial clearance and for the control of dissemination of *S*. Typhimurium to extraintestinal sites, and this is not necessarily related to the anticipated development of the disease, but it is possible that all these factors could allow the bacteria to survive longer inside the cells and generate an inflammatory atmosphere that promotes the onset of IBD.

Some studies have demonstrated that MDP induces autophagy in dendritic cells, a process that needs correct NOD2 signaling, which in turn requires mainly the proper function of ATG16L1. These proteins are required for the correct function of NOD2 and antigen presentation (125). Mutation in this gene generates a malfunction in the autophagy process in dendritic cells, which indicates an aberrant bacterial trafficking and failure to produce antigen presentation on MHC-II molecules, which in turns promote the generation of antigen-specific, effector CD4^+^ T cells (125). All these defects may allow bacteria to survive longer inside dendritic cells, avoiding lysosomal degradation for extended time (126, 127) and to provide a mechanism for the persistence of the pathogen and in consequence the persistent inflammation. Beside this, it has been reported that *S*. Typhimurium employs dendritic cells expressing CCR7 as a pathway to migrate from the intestine to MLNs (127).

The above background suggests that during an infection with *S*. Typhimurium, the bacterial virulence factors and the defective processes in susceptible individuals, such as those described in IBD patients, could generate a persistence of the bacteria in dendritic cells, which would generate a continuous secretion of pro-inflammatory cytokines and an environment of inflammation, which means that an infection with *S*. Typhimurium in these patients could have a double effect, being more permissive to the infection caused by this intracellular bacteria. Because of this, we propose that an infection with *S*. Typhimurium could anticipate the onset of the disease, due to the atmosphere of inflammation that it generates. Furthermore, the infection with *S*. Typhimurium changes the composition of the microbiota and the permeability of the epithelial barrier, which could be a trigger for the disease in susceptible individuals, given that these changes modify the production of cytokines and SCFAs, produce an influx of neutrophils and persistence infection of dendritic cells and, in consequence, generate an abnormal inflammatory environment. All this, combined with a genetic susceptibility, will impair the recognition of pathogens, the autophagy, tissue repair, and bacterial clearance, generating an inflammatory condition at the intestinal epithelium.

## Concluding Remarks

In this review, we discussed several microbial, cellular, and genetic alterations described so far in IBD patients, and related these defects with the infection caused by *S*. Typhimurium. It is possible that *S*. Typhimurium infection could trigger chronic inflammation in individual carrying one or more of the defects associated with IBD, given the inability of these patients to properly clear bacteria in the intestine. Moreover, *S*. Typhimurium has an important arsenal of virulence factors to invade host cells in the intestinal epithelium and lamina propria that the normal microbiota is not able to reach. Additionally, it is known that this bacterium can cause persistent infection in human and in mice, suggesting that in patients displaying one or more genetic defects that predispose to IBD development; it is possible that they are much more susceptible to be infected by *S*. Typhimurium and cause a persistent infection. Permanent infection of cell with *S*. Typhimurium could promote secretion of pro-inflammatory cytokines by infected cells, generating an inflammatory environment in the intestinal layers, promoting changes in the microbiota and promoting chronic diseases. It would be relevant to evaluate whether IBD patients are chronic carriers of *S*. Typhimurium in the intestine.

Supporting the hypothesis raised in this review, it has been described that IBD patients have a deregulated immune response in the intestine, which is reflected for instance by the secretion of IgG instead of IgA, resulting in an inflammatory response against their own microbiota (56). This loss of tolerance to the microbiota could be determining factor to the infection with an invasive bacterium as *S*. Typhimurium, which could promote a permanent inflammatory response in the intestine, which in turn could bias the humoral immune response to an IgG type to other bacteria, as the intestinal microflora. It has been described that in IBD patients the commensal microbiota has different phyla proportions in comparison to a healthy person, having less amount of *Bacteroidetes* and *Firmicutes* (beneficial bacteria) (56, 66, 67). This allows less competition to pathogenic bacteria, making them more invasive. On the other hand, in these patients, the epithelial barrier is impaired because of inflamed epithelial cells, continuous secretion of pro-inflammatory cytokines, and disrupted tight junctions (54). It is possible that all these alterations could be due to *S*. Typhimurium infection, which results in an increased inflammation in susceptible patients (44, 46, 54).

It is known that *S*. Typhimurium can produce an inflammatory environment due to virulence proteins coded by SPIs, which improve its fitness over intestinal microbiota and, thereby, to reach and invade the epithelium (79). Therefore, the inflammation would not be detrimental for this kind of invasive pathogen and conversely it might be facilitating growth (41, 89, 128, 129). Even more, it is possible that through this mechanism, *S*. Typhimurium can accelerate the development of the disease in people with genetic susceptibility. Due to the factors mentioned above, it is possible that in IBD patients will be more susceptible to suffer a more aggressive infection by *S*. Typhimurium or even to develop a persistent infection, due to the baseline inflammation and impaired intestinal environment. Therefore, the development of new methods that could prevent early colonization with this pathogen in this type of patients is a challenge for future research.

## Author Contributions

All authors listed have made substantial, direct, and intellectual contribution to the work and approved it for publication. The authors are grateful to Pamela A. Nieto, Ph.D., who edited this manuscript.

## Conflict of Interest Statement

The authors declare that the research was conducted in the absence of any commercial or financial relationships that could be construed as a potential conflict of interest.
